# Effect of Adding Gadolinium Oxide Promoter on Nickel Catalyst over Yttrium-Zirconium Oxide Support for Dry Reforming of Methane

**DOI:** 10.3390/ma16031158

**Published:** 2023-01-29

**Authors:** Salwa B. Alreshaidan, Ahmed Al-Fatesh, Mahmud S. Lanre, Yousef M. Alanazi, Ahmed A. Ibrahim, Anis H. Fakeeha, Fahad Albaqi, Khalid Anojaidi, Abdulaziz Bagabas

**Affiliations:** 1 Department of Chemistry, Faculty of Science, King Saud University, P.O. Box 800, Riyadh 11451, Saudi Arabia; 2 Chemical Engineering Department, College of Engineering, King Saud University, P.O. Box 800, Riyadh 11421, Saudi Arabia; 3 King Abdullah City for Atomic & Renewable Energy, Energy Research & Innovation Center (ERIC) in Riyadh, Riyadh 11451, Saudi Arabia; 4 President Office, King Abdulaziz City for Science and Technology (KACST), P.O. Box 6086, Riyadh 11442, Saudi Arabia

**Keywords:** dry reforming of methane, carbon resistance, gadolinium oxide, yttrium-zirconium oxide, nickel catalyst

## Abstract

The dry reforming of methane (DRM) was studied for seven hours at 800 °C and 42 L/(g·h) gas hourly space velocity over Ni-based catalysts, promoted with various amounts of gadolinium oxide (x = 0.0, 1.0, 2.0, 3.0, 4.0, and 5.0 wt.%) and supported on mesoporous yttrium-zirconium oxide (YZr). The best catalyst was found to have 4.0 wt.% of gadolinium, which resulted in ∼80% and ∼86% conversions of CH_4_ and CO_2_, respectively, and a mole ratio of ∼0.90 H_2_/CO. The addition of Gd_2_O_3_ shifted the diffraction peaks of the support to higher angles, indicating the incorporation of the promoter into the unit cell of the YZr support. The Gd_2_O_3_ promoter improved the catalyst basicity and the interaction of NiO with support, which were reflected in the coke resistance (6.0 wt.% carbon deposit on 5Ni+4Gd/YZr; 19.0 wt.% carbon deposit on 5Ni/YZr) and the stability of our catalysts. The Gd_2_O_3_ is believed to react with carbon dioxide to form oxycarbonate species and helps to gasify the surface of the catalysts. In addition, the Gd_2_O_3_ enhanced the activation of CH_4_ and its conversion on the metallic nickel sites.

## 1. Introduction

Energy is fundamental to modern economies, and it is anticipated that its demand will continue to rise for many years to come [1,2,3,4]. The bulk of this energy is derived from fossil fuel, which emits the two most common greenhouse gases (CH_4_ and CO_2_). For centuries, the burning of hydrocarbons has increased the atmospheric CO_2_ concentration. Methane remains in the atmosphere for much less time than carbon dioxide, but it is more potent in the greenhouse effect [5,6,7]. The concentration of these two greenhouse gases is steadily rising, causing global warming, which harms biodiversity and the ecosystem.

Developing active, selective, energy-efficient heterogeneous catalytic processes is key to a sustainable future because heterogeneous catalysis is at the center of the chemicals and energy industries. The increased urgency of catalyst development for key processes, for instance, biomass upgrading, CO_2_ reduction, water splitting, and light alkanes activation, is due to the soaring demand for energy, chemical products, and food and the rise in anthropogenic CO_2_ emissions worldwide. As a result, several investigators have performed deep research on various processes. Zada et al. investigated photocatalytic H_2_ generation and pollutant withdrawal using g-C_3_N_4_ with SnO_2_ [8]. Alternatively, Hayat et al. explored the photocatalytic conversion of CO_2_ reduction and hydrogen production by water splitting, employing conjugated co-monomer 3, 6-dibromopyridazine (DBP) integrated into the triazine framework of polymer carbon nitride [9].

The dry reforming of methane (DRM) process converts these greenhouse gases to produce syngas (a mixture of H_2_ and CO), deplete their concentration in the atmosphere, and mitigate their effect on global warming [10,11]. The syngas serves as feedstock for the Fischer-Tropsch (FT) method to generate valuable chemicals like methanol [12,13].

Nickel-based catalysts are promising because of their relative cheapness and availability compared to noble metals [5]. The drawbacks of nickel-based catalysts deal with sintering and coke deposition arising from CH_4_ cracking and CO disproportionation side reactions [14]. Catalyst promoters and relevant supports help to reduce carbon formation [15,16,17,18]. The redox properties of supports correlate with their capabilities to remove carbon deposition during the DRM reaction [19]. The reaction between the formed carbon and the lattice oxygen on the surface of the support increases the ability of CO_2_ adsorption and dissociation, as well as the re-oxidation of the support [7]. Catalyst support alters the catalyst’s active surface area and acid-base characteristics and influences the catalytic activity of metals [20]. Bioethanol steam reforming using Rh-Ni catalysts, supported on a yttrium-modified γ-Al_2_O_3_ carrier, gave high hydrogen yield and stability, owing to the increase of basic properties of the support [21]. With its distinct chemical and thermal properties, yttrium oxide (Y_2_O_3_) can function as either a promoter or support. The steam reforming of ethanol over a Ni/Y_2_O_3_ catalyst produced a high percentage of hydrogen yield because of the enhanced activity, stability, and ease of reduction made by the Y_2_O_3_-supported Ni catalyst [21].

Zirconia is a good active metal carrier because of its heat stability and unique properties, such as reduction-oxidation and acid-base characteristics [22,23,24,25,26]. Santamaria et al. [27] proved that ZrO_2_ was suitable to support nickel because it produced limited coke with low-temperature carbon combustion. Supporting nickel catalyst over yttria-zirconia for DRM reduced the formation of carbonaceous deposits and lengthened the life span of the catalyst [7]. Alkali earth and alkali with rare earth metal promoters have been used to boost the activity and stability of nickel-based catalysts [21]. Ni catalyst was doped with a small amount of noble metal to improve the catalytic performance in a DRM reaction [21].

Al-Fatesh et al. [28] studied the effect of gadolinium Gd_2_O_3_ as a promoter for Ni/Y_2_O_3_ catalyst in the DRM to produce hydrogen. It was found that 1 wt.% of Gd_2_O_3_ reduced carbon deposition significantly when compared to an un-promoted catalyst. Gd_2_O_3_ helped to modify catalyst textural features by enhancing the dispersion of Ni metal over the support [29,30]. At high calcination temperatures, the Gd_2_O_3_ promoter tended to maintain its textural property by improving the catalyst basicity [28]. The higher the catalyst basicity, the better the CO_2_ adsorption and dissociation and the less the carbon deposition [31,32]. Because of the synergistic effect of Gd_2_O_3_ and Ni, the activity of the Gd_2_O_3_-promoted catalyst could be increased with less deactivation [33]. Ni particles outside carbon nanotubes (CNTs) are more susceptible to carbon deposition than those embedded inside the CNTs. Gd_2_O_3_-promoted Ni/Y_2_O_3_ underwent a few-walled CNTs formation so that CNTs formation did not significantly affect catalyst activity [34].

This research aims to investigate the effects of the Gd_2_O_3_ promoter on the activity and stability of the yttrium-zirconium oxide-supported nickel catalyst. Furthermore, the optimization of Gd_2_O_3_ loading for the best catalytic performance is another target for this work.

## 2. Materials and Methods

### 2.1. Materials

Gadolinium nitrate hexahydrate [Gd(NO_3_)_3_.6H_2_O; 99.9% trace metals basis; Ventron, Alfa Produkte]; mesoporous 8.0 wt.% yttria-stabilized zirconia (meso-8Y_2_O_3_-ZrO_2_; meso-YZr; Anhui-Elite, Hefei, China); and nickel nitrate hexahydrate [Ni (NO_3_)_2_.6H_2_O, 98%, Alfa Aesar] were used as received. Ultrapure water was obtained via a Milli-Q water purification system (Millipore).

### 2.2. Synthesis of Catalysts

Both the nickel and gadolinium nitrates were loaded over meso-YZr support by the dry impregnation method. The required amount of nickel nitrate hexahydrate to give 5.0 wt.% of Ni and the required amount of gadolinium nitrate hexahydrate to give 0.0, 1.0, 2.0, 3.0, 4.0, or 5.0 wt.% of Gd_2_O_3_ were mixed and ground with the support, followed by the addition of drops of ultrapure water to obtain a green paste. Upon mechanical stirring, this paste was dried and ground. Mechanical stirring and water addition were repeated three times. The mixtures were calcined for three hours at 600 °C with a temperature ramp of 3.0 °C/min.

### 2.3. Catalyst Activity

DRM experiments were performed at 800 °C under ambient pressure. A tubular stainless-steel reactor (i.d. = 0.009 m; length = 0.3 m) was used. An amount of 0.1 g of the catalyst was used for catalytic testing. The temperature was measured using a sheathed stainless-steel K-type thermocouple, which was placed axially at the center of the catalyst bed. The catalyst was reduced for one hour at 700 °C with a H_2_ flow prior to the reaction. During the experiments, methane, carbon dioxide, and nitrogen gases were mixed in a 3:3:1 volume ratio. This mixture was used as a reactant feed with a space velocity of 42 L/h/g_cat_. The effluent gas was connected to an online GC, which was equipped with a thermal conductivity detector (TCD) to analyze its composition. CH_4_ and CO_2_ conversions and syngas ratio were calculated, as shown below:

(1) $${\mathrm{CH}}_{4}\mathrm{Conversion}:=\frac{{\mathrm{CH}}_{4,\mathrm{in}}-{\mathrm{CH}}_{4,\mathrm{out}}}{{\mathrm{CH}}_{4,\mathrm{in}}}\times 100\%$$

(2) $${\mathrm{CO}}_{2}\;\mathrm{conversion}\;\left(\%\right)=\frac{{\mathrm{CO}}_{2,\mathrm{in}}-{\;\mathrm{CO}}_{2,\mathrm{out}\;}}{{\mathrm{CO}}_{2,\mathrm{in}}}\times 100$$

(3) $$\frac{H_{2}}{\mathrm{CO}}=\frac{{\mathrm{moles}\;\mathrm{of}\;H}_{2}\mathrm{produced}}{\mathrm{moles}\;\mathrm{of}\;\mathrm{CO}\;\mathrm{produced}\;}$$

### 2.4. Catalyst Characterization

X-ray diffraction (XRD) patterns of the catalysts were recorded on a Thermo Fisher diffractometer equipped with Cu Kα X-ray radiation and operated at 40 mA and 40 kV. The isotherms of nitrogen physisorption, depending on the Brunauer–Emmett–Teller method (BET), were determined using a Micromeritics Tristar II 3020 surface area and porosity analyzer at −196 °C after outgassing the samples at 200 °C for three hours to remove any adsorbed gases or vapors. The distributions of pore size of the samples were analyzed from the adsorption of isotherms using the Barrett–Joyner–Halenda (BJH) model. Hydrogen temperature-programmed reduction (H_2_-TPR) and carbon dioxide temperature-programmed desorption (CO_2_-TPD) analyses of the freshly synthesized catalysts were performed on a Micromeritics Auto Chem II 2920. The analyses were conducted over a temperature range of 50–800 °C and 40 mL/min flow of 10% H_2_/Ar mixture for the TPR analysis and 10% CO_2_/He mixture for CO_2_-TPD measurement, respectively. The coke formation and the amount of carbon deposit on the surface of the spent catalysts were assessed by a thermogravimetric analyzer (Shimadzu-TGA). The deposited carbon was burned in an air atmosphere by heating the samples up to 1000 °C at a rate of 10 °C/min and recording the weight loss. The morphology of the catalysts was examined using a high-resolution transmission electron microscope (HRTEM model: JEM-2100 F, JEOL; Akishima, Tokyo, Japan) and a field emission scanning electron microscope (FE-SEM, 7100F (JEOL; Tokyo, Japan) equipped with energy-dispersive X-ray spectroscopy (EDX) for surface elemental analysis.

## 3. Results and Discussion

### 3.1. Nitrogen Physisorption Analysis

**Figure 1:**
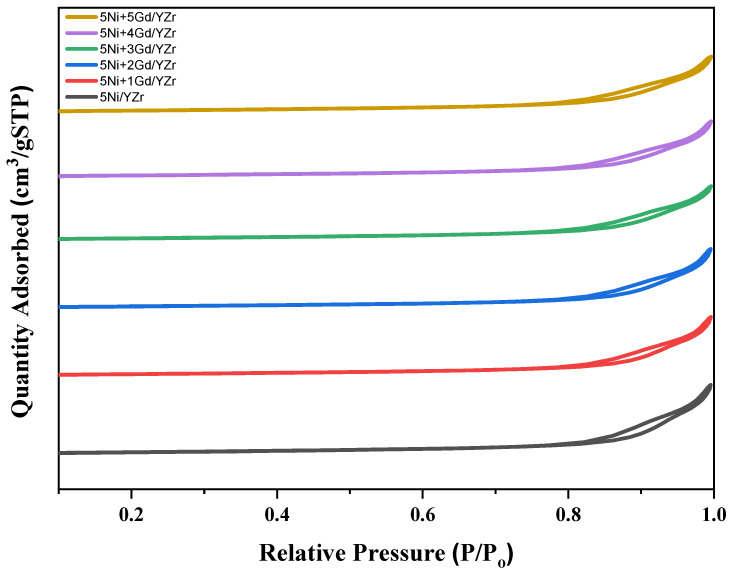
Nitrogen physisorption isotherms of the fresh catalysts.

The nitrogen isotherms were of type IV, as shown in Figure 1. Table 1 shows the BET surface area (S_BET_), pore volume (P_v_), and average pore diameter (P_d_) for all the catalysts. The surface area of the unprompted catalyst (5Ni/YZr) displayed the highest specific surface area. After the addition of the Gd_2_O_3_ promoter, the specific surface area decreased slightly to a range of 26.0–27.0 m^2^/g. For all catalysts, an increase of the relative pressure at 0.8 was observed. H3 hysteresis loops were exhibited in all the catalysts, indicating the presence of aggregates of plate-like particles that resulted in slit-shaped pores. Table 1 discloses that the surface area of the Gd_2_O_3_-promoted catalysts was primarily unaffected by the variation of Gd_2_O_3_ promoter loadings, implying that the Gd_2_O_3_ particles diffused inside the pores of the support.

### 3.2. Hydrogen Temperature-Programmed Reduction (H_2_-TPR)

**Figure 2:**
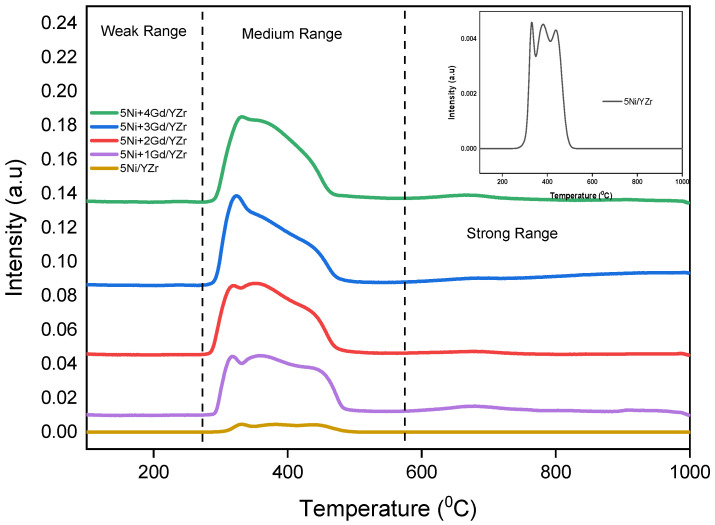
H_2_-TPR profiles of the fresh catalysts (inset of figure: H_2_-TPR of 5Ni/YZr added for clarity because of its very weak TCD intensity).

The TPR profile of the 5Ni/YZr catalyst is shown in the inset Figure 2 (With its very low intensity, this TPR profile diminished when combined with the other profiles, and thus, it is shown in the inset). The reduction peak appeared at a moderate-temperature region of around 300–500 °C with a broad peak and three maxima, corresponding to the reduction of bulk NiO. Generally, this kind of reduction is a characteristic feature of stoichiometric NiO [30]. The appearance of these reduction peaks in the moderate-temperature region indicates a good interaction between the support and the NiO. The absence of reduction peaks below 300 °C indicates that the unprompted catalysts had neither free NiO species nor weakly interacted NiO species with the support. Figure 2 depicts the TPR profiles of the Gd_2_O_3_-promoted catalysts, where the reduction peaks in the temperature range of 300–500 °C, which indicates relatively easy and high reducibility of the NiO phases. With the increase in Gd_2_O_3_ loading, the broad peak in the temperature range of 300–500 °C shifted progressively toward a lower temperature, and a small peak below 300 °C emerged, owing to the formation of alloy at the NiO/Gd_2_O_3_ interfaces [35]. In addition, for the Gd_2_O_3_-promoted catalysts, the TPR profiles showed broad, low-intensity peaks between 600 and 800 °C, which could be attributed either to the reduction of Gd_2_O_3_ [36] or to the reduction of strongly interacted NiO species with the meso-YZr support because of the presence of the promoter [37].

### 3.3. Carbon Dioxide Temperature-Programmed Desorption (CO_2_-TPD)

**Figure 3:**
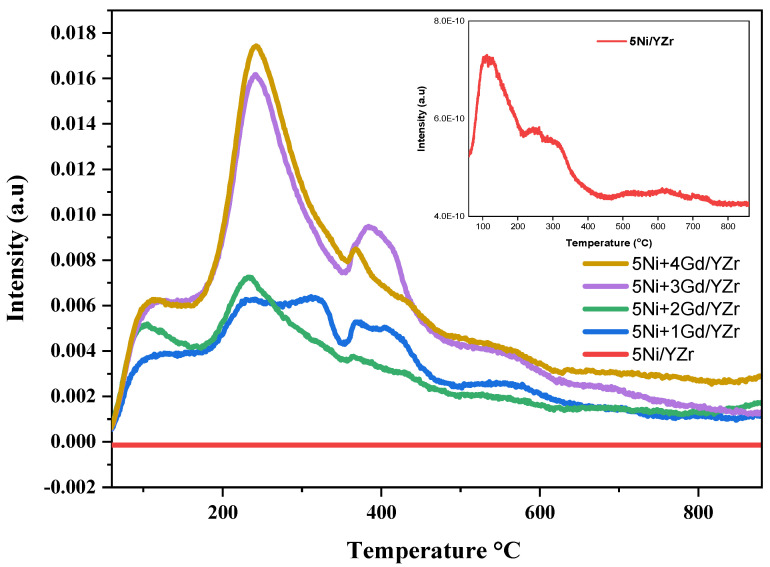
CO_2_−TPD profiles of the fresh catalysts (inset Figure: CO_2_-TPD of 5Ni/YZr added for clarity because of its very weak TCD intensity).

Because the acidic support increases the coke deposition, the researchers focused on nickel catalysts, supported or promoted by metal oxides with strong Lewis basicity. The basicity of the un-promoted and Gd_2_O_3_-promoted fresh catalysts was estimated by CO_2_-TPD experiments, as illustrated in Figure 3. For Gd_2_O_3_-promoted catalysts, desorption peaks with maxima centered around 200–300 °C were associated with both weak Bronsted basic sites, such as surface OH^−^ groups, and medium-strength Lewis base sites, while the un-promoted catalyst showed desorption peaks at maxima centered around 100–200 °C associated only to weak Bronsted basic sites. In general, the CO_2_-TPD profiles had similar temporal features. However, when increasing the Gd_2_O_3_ loading, the intensity of the peaks in CO_2_-TPD profiles increased, implying the increase of catalysts’ basicity. The basicity of the Gd_2_O_3_-promoted catalysts was moderate because of the appearance of the desorption peaks in the moderate-temperature region between 200 and 500 °C. The relatively high basicity of the 5Ni+4Gd/YZr catalyst favored efficient CO_2_ adsorption and dissociation, which helped to reduce carbon deposits and catalyst deactivation [24,25,26].

### 3.4. XRD Analysis

**Figure 4:**
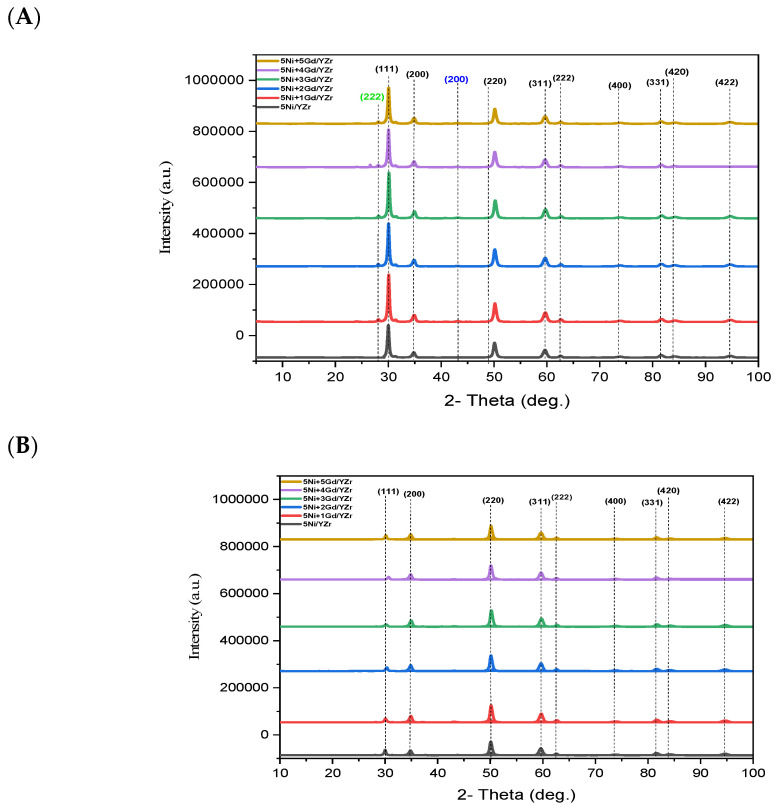
The XRD patterns of (**A**) the fresh catalysts and (**B**) the spent catalysts (black labels for mesoporous YZr support, green label for Gd_2_O_3_promoter, and blue label for NiO).

The XRD patterns of the fresh 5Ni+xGd/YZr (x = 0, 1, 2, 3, 4, or 5) catalysts are shown in Figure 4A. The XRD patterns of 5Ni/YZr and Gd_2_O_3_-promoted catalysts displayed peaks at 2θ of ~30, ~35, ~50, ~60, ~63, ~74, ~82, ~84, and ~94°, which refer, respectively, to the (111), (200), (220), (311), (222), (400), (331), (420), and (422) crystallographic planes of the cubic phase of yttria-stabilized zirconia (JCPDS No. 49-1642). Furthermore, the peak at 2θ of ~43° (200 crystallographic plane) could be ascribed to the cubic phase of nickel oxide (PDF 00-044-1159). The peak at 2θ ~28° could be ascribed to the cubic phase of gadolinium oxide (JCPDS No. 12-0797) for the crystallographic phase with Miller indices (222). The addition of the Gd_2_O_3_ promoter shifted the peaks of YZr support slightly to a higher 2θ angle; i.e., it caused a slight reduction in the d-spacing parameter, implying the incorporation of Gd_2_O_3_ in the lattice of the YZr support, as shown in Table 2.

The XRD patterns of the spent 5Ni+xGd/YZr (x = 0, 1, 2, 3, 4, or 5) catalysts are shown in Figure 4B. The XRD patterns of 5Ni/YZr and Gd_2_O_3_-promoted catalysts displayed peaks at 2θ of ~30, ~35, ~50, ~60, ~63, ~74, ~82, ~84, and ~94°, which refer, respectively, to the (111), (200), (220), (311), (222), (400), (331), (420), and (422) crystallographic planes of the cubic phase of yttria-stabilized zirconia (JCPDS No. 49-1642). As shown in Table 3, the intensity and the broadness of the peaks were reduced in comparison to those of the fresh catalysts. This observation could be attributed to the deposition of carbon, where a higher amount of deposited carbon yielded a larger d-spacing, as confirmed by TGA results. Moreover, the absence of NiO diffraction peak in the patterns of the spent catalysts might be due to its reduction to metallic Ni, which was incorporated in the multi-walled carbon nanotubes. The disappearance of the Gd_2_O_3_ diffraction peak would be ascribed to its conversion to Gd_2_O_2_CO_3_, as illustrated in the plausible mechanism section below.

**Figure 5:**
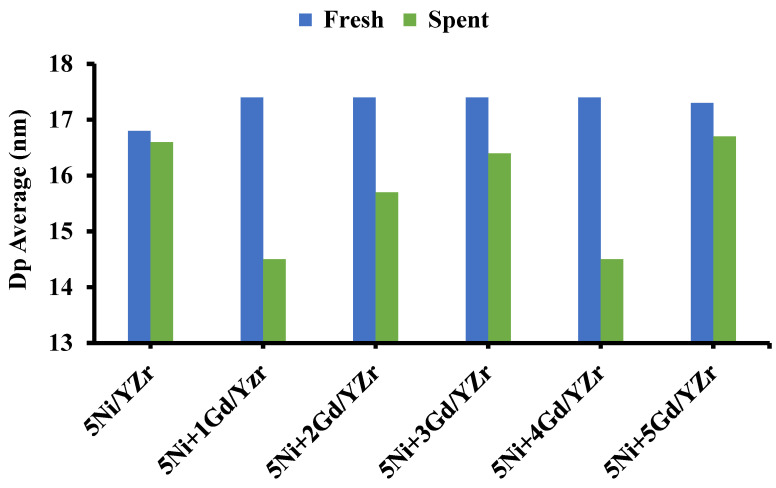
Average crystallite size for the fresh and spent catalysts.

Scherrer’s equation was utilized to assess the crystallite size:

(4) $$D_{p}=K*\lambda /\beta \;\cos\theta$$

where D_p_ is the crystallite size in nanometers; λ is the X-ray wavelength (0.15406 nm); β is the full width at half maximum of the diffraction peak of the sample; *K* is the shape factor, which is 0.94; and θ is the diffraction angle in degrees. The crystallite sizes of all fresh yttria-stabilized zirconia-supported Ni catalysts (Gd_2_O_3_ wt%: 0.0, 0.1, 0.2, 0.3, 0.4, 0.5) were determined from XRD patterns using the most intense peaks at ~30 and 50°, as shown in Figure 5. The smallest crystallite size (16.80 nm) was observed for the un-promoted catalyst. Upon incorporating the Gd_2_O_3_ promoter, the crystallite size increased to 17.3 nm for 5Ni+4Gd/YZr and 5Ni+5Gd/YZr and to 17.4 nm for 5Ni+1Gd/YZr, 5Ni+2Gd/YZr, and 5Ni+3Gd/YZr. This finding could be due to the higher incorporation of Gd_2_O_3_ into YZr support for 5Ni+4Gd/YZr and 5Ni+5Gd/YZr. On the other hand, the smallest crystallite size (14.5 nm) was found for 5Ni+1Gd/YZr and 5Ni+4Gd/YZr spent catalysts, followed by 5Ni+2Gd/YZr (15.7 nm), 5Ni+3Gd/YZr (16.4 nm), un-promoted catalyst (16.6 nm), and 5Ni+5Gd/YZr (16.7 nm). However, we could not explain this trend.

### 3.5. Catalytic Activity

**Figure 6:**
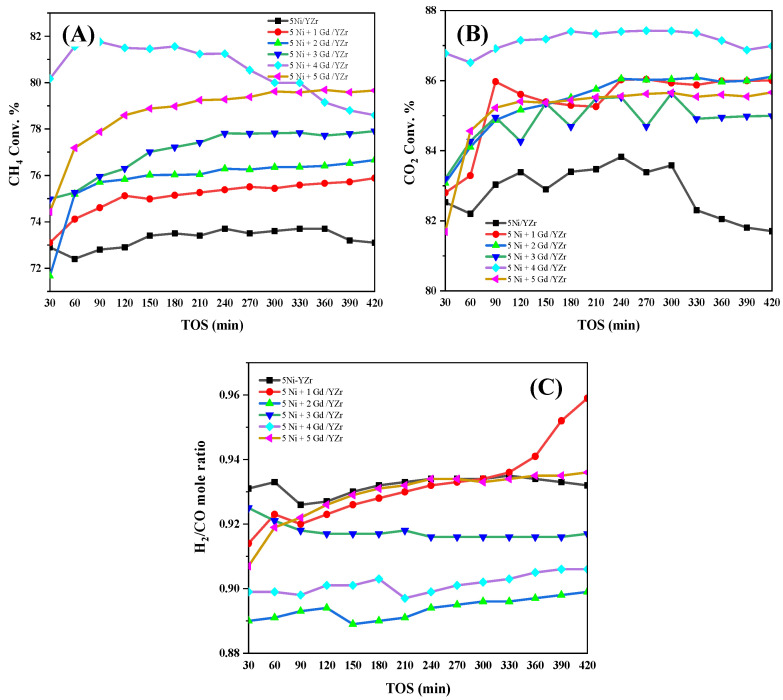
The conversions of (**A**) CH_4_, (**B**) CO_2_, and (**C**) H_2_/CO mole ratio at 800 °C, one atmosphere, and GHSV = 42 L/h/g_cat_.

The performance of the catalysts is presented in Figure 6. The reaction was performed at 800 °C and 1.0 atm and for a duration of seven hours. The catalyst activity was expressed in terms of CH_4_ and CO_2_ conversions and the H_2_/CO mole ratio. The general trend of results showed a decrease in the conversions along with TOS due to deactivation by carbon deposition, as confirmed by the TGA results. The 5Ni+4Gd/YZr catalyst was found to be the best in DRM, where it resulted in ∼80% and ∼86% conversions of CH_4_ and CO_2_, respectively, and a mole ratio of ∼0.90 H_2_/CO. The methane conversion profile of the catalysts (5Ni+xGd/YZr, x = 0, 1, 2, 3, 4, or 5) showed an increasing trend from 0.0 wt.% Gd_2_O_3_ up to 4.0 wt.% Gd_2_O_3_ and then tended to decline at 5.0 wt.% Gd_2_O_3_. For all catalysts, the CH_4_ conversion was lower than that of CO_2_. This observation could be linked to the reverse water gas shift (RWGS) reaction, which consumed CO_2_ alongside the main reaction. The H_2_/CO ratio profile showed a declining tendency, which could be ascribed to the occurrence of the RWGS reaction, where the produced H_2_ was consumed by the CO_2_ in the feed to generate CO and thus caused a drop in H_2_/CO mole ratio. A comparison between our best catalyst, 5Ni+4Gd/YZr, and other similar catalysts in terms of the conversions of CH_4_ and CO_2_, as well as H_2_/CO mole ratio, is shown in Table 4.

### 3.6. Transmission Electron Microscope (TEM)

**Figure 7:**
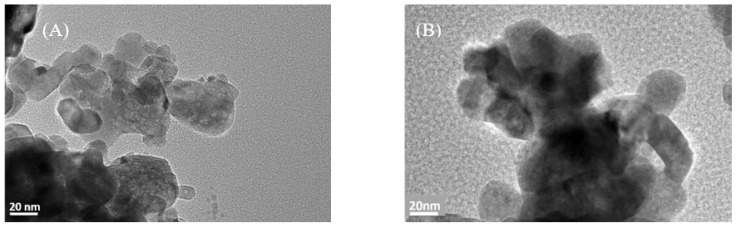
TEM micrograph images at magnification of 200,000× for (**A**) fresh and (**B**) spent 5Ni+4Gd/YZr catalysts.

TEM images of both fresh and spent 5Ni+4Gd/YZr catalysts are shown in Figure 7. The fresh and spent catalyst particles were agglomerated. The TEM images do not show the filamentous carbon deposits on the spent 5Ni+4Gd/YZr catalyst. This observation could be attributed to the even distribution of carbon deposits on the surface of the catalyst and to the carbon-resistance feature of our catalyst, as discussed later on the “plausible mechanism”. Moreover, the TEM images indicate no significant change in particle size of the spent catalyst in comparison to the fresh one, indicating the sintering resistance feature of our catalyst. Such observation is consistent with the crystallite size determined from the XRD pattern.

**Figure 8:**
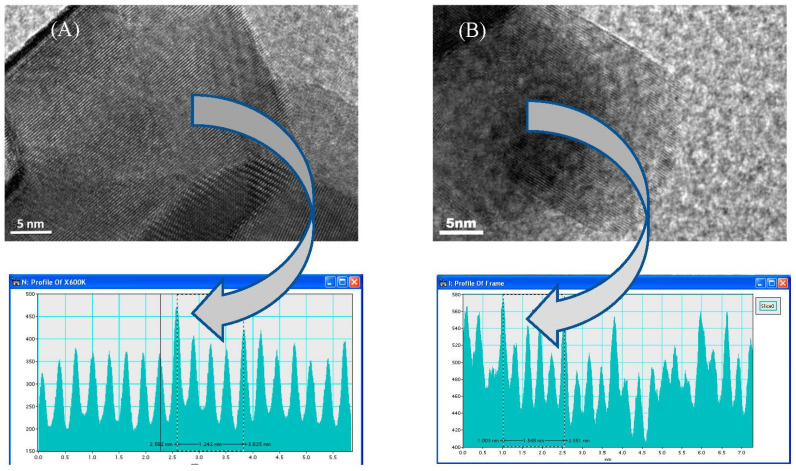
HRTEM micrograph images at magnification of 600,000× for (**A**) fresh and (**B**) spent 5Ni+4Gd/YZr catalysts.

The high-resolution TEM (HRTEM) of the fresh and spent 5N+4Gd/YZr catalysts showed parallel lattice plane fringes of the YZr support. The d-spacing value was calculated from the corresponding distance between the lattice plane fringes, as shown in Figure 8 and Table 5, where the calculated d-spacing values were similar to those of the bulk YZr support. These results also indicated the good crystallinity of our support without affecting the d-spacing values for the 400 and 411 crystallographic planes by loading NiO and Gd_2_O_3,_ as well as the carbon deposition.

### 3.7. Scanning Electron Microscope (SEM)

**Figure 9:**
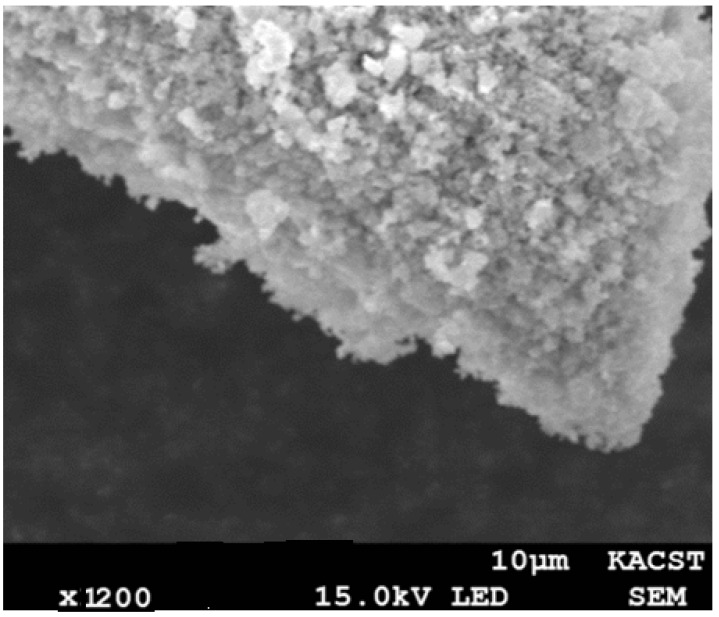
SEM image of the fresh 5Ni+4Gd/YZr catalyst.

The SEM technique was used to investigate the morphology of the catalysts. Figure 9 shows the SEM image of the fresh sample of the best catalyst (5Ni+4Gd/YZr), where agglomerated particles were observed.

**Figure 10:**
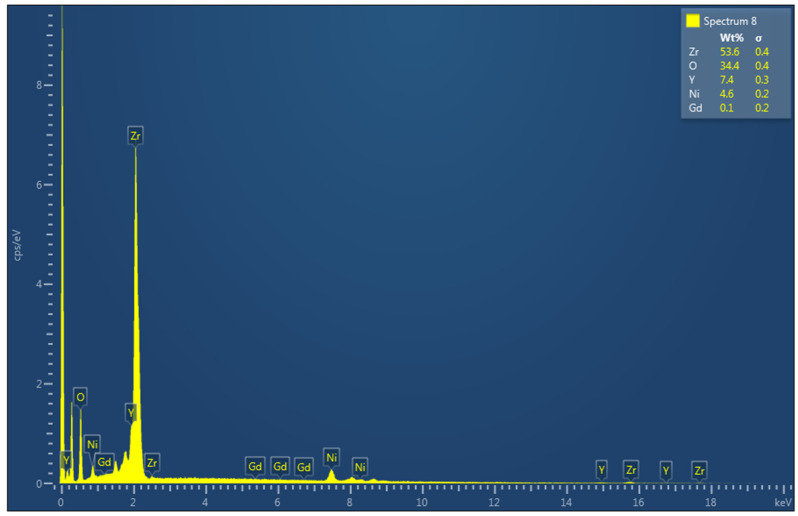
The EDX spectrum of the fresh 5Ni+4Gd/YZr catalyst.

Figure 10 displays the EDX analysis of the best catalyst, where all the elements expected to be on the surface were detected qualitatively, implying the success of our preparation method.

### 3.8. Thermogravimetric Analysis (TGA) of the Spent Catalyst

**Figure 11:**
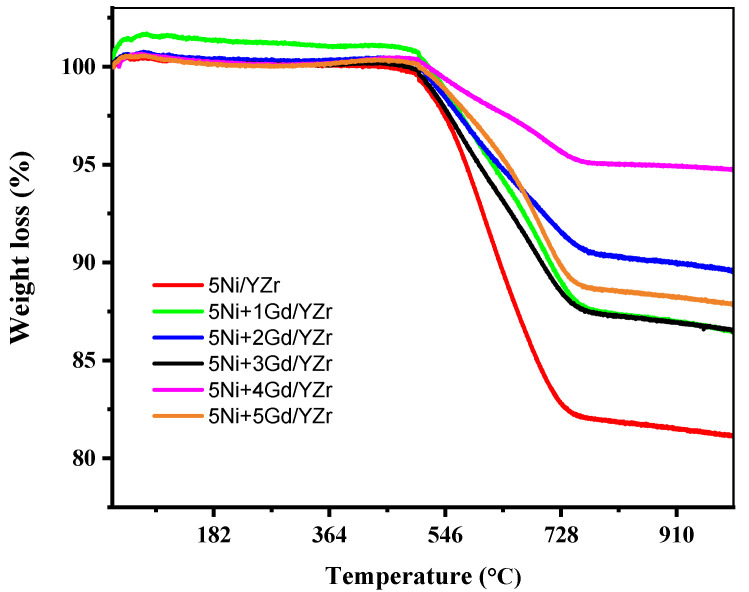
TGA plot of the spent catalysts after seven hours of time-on-stream.

Based on the TGA plot (Figure 11), all the spent catalysts displayed weight loss in the temperature range of 500–1000 °C. The 5Ni/YZr catalyst contained ~19 wt.% of carbon deposit, while the Gd_2_O_3_-promoted catalysts had ~6–14 wt.% of carbon deposit, depending on Gd_2_O_3_ loading. The 5Ni+4Gd/YZr catalyst produced the least carbon deposition of ~6 wt.%. This observation indicates that Gd_2_O_3_ not only promoted the reaction performance of the catalysts but also contributed to increasing the coke resistance of the catalysts. The amount of carbon deposit on the Gd_2_O_3_-promoted catalysts (1.0, 2.0, 3.0, and 5.0 Gd_2_O_3_ wt.%) was relatively close to each other. The promotional effect and coke resistance of the Gd_2_O_3_-promoted catalyst could be explained by the increased adsorption and activation of CO_2_ on Gd_2_O_3_ sites as carbonates (Gd_2_O_2_CO_3_) [40]. Furthermore, Gd_2_O_3_ also increased the CH_4_ activation and conversion on the metallic nickel sites.

### 3.9. Plausible Mechanism

It is well-known that zirconium oxide (ZrO_2_) facilitates the decomposition of carbon dioxide into carbon monoxide and oxygen radical, owing to the oxygen vacancy in ZrO_2_ support, as shown in Equation (5):

(5) $${\mathrm{CO}}_{2}+{⎕}_{\mathrm{Zr}}\;\to \;{\mathrm{CO}}^{*}+O_{\mathrm{Zr}}$$

where $⎕{}_{\mathrm{Zr}}$ and $O_{\mathrm{Zr}}$ are for oxygen vacancy and oxygen on the surface of ZrO_2_ support, respectively.

Moreover, carbon monoxide could be created by the decomposition of the bicarbonate intermediate, as displayed in Equations (6) and (7):

(6) $$CO_{2}{|}_{Zr}+{\mathrm{OH}}_{Zr}\;\to \;{\mathrm{HCO}}_{3}|{}_{Zr}$$

(7) $${\mathrm{HCO}}_{3}|{}_{Zr}+⎕{}_{\mathrm{Zr}}+*\;\to {\;\mathrm{CO}}^{*}+{\mathrm{OH}}_{\mathrm{Zr}}+O_{\mathrm{Zr}}$$

where ${\mathrm{CO}}_{2}|{}_{\mathrm{Zr}}{\;\mathrm{and}\;\mathrm{OH}}_{\mathrm{Zr}}$ are, respectively, adsorbed carbon dioxide and hydroxyl species on the ZrO_2_ surface.

Yttria-stabilized ZrO_2_ has more oxygen vacancies and possesses basic sites, and therefore, it enhances the decomposition of carbon dioxide according to Equation (5) [37].

Incorporating of Gd_2_O_3_ promoter into the catalyst could facilitate the formation of Gd_2_O_2_CO_3_ on the catalyst surface, owing to the interaction of basic Gd_2_O_3_ with the acidic CO_2_ as per Equation (8) [39]:

(8) $${\mathrm{Gd}}_{2}O_{3\;}+{\mathrm{CO}}_{2}\to \;{\mathrm{Gd}}_{2}O_{2}{\mathrm{CO}}_{3}$$

The Gd_2_O_2_CO_3_ surface species would react promptly with the adsorbed CH_x_ species, which emerged from the dissociation of methane on nickel metallic active sites, as illustrated in Equation (9):

(9) Gd2O2CO3+CHx → 2CO*+Gd2O3+x2H2

In addition, the formed Gd_2_O_2_CO_3_ participated in the gasification of the deposited carbon, resulting from the decomposition of methane, and hence, in the regeneration of the metallic nickel active sites for hydrogen production, as shown in Equation (10):

(10) $${\mathrm{Gd}}_{2}O_{2}{\mathrm{CO}}_{3}+C\;\to \;2\mathrm{CO}+{\mathrm{Gd}}_{2}O_{3}$$

The Gd_2_O_2_CO_3_ species also has the capability to react with the adsorbed hydrogen atom, produced via methane decomposition, to generate the Gd_2_O_3_, CO, and an active surface hydroxyl group, as displayed in Equation (11):

(11) $${\mathrm{Gd}}_{2}O_{2}{\mathrm{CO}}_{3}+H^{*}\;\to {\mathrm{Gd}}_{2}O_{3}+\mathrm{CO}+{\mathrm{OH}}^{*}$$

Thus, as per the above-suggested reaction scenario, we think that the interfacial areas among Gd_2_O_2_CO_3_ and metallic nickel would be the most active sites for DRM, where carbon dioxide activation is improved by the formation of carbonate species, which, in turn, urges and accelerates the decomposition of methane [39]. Moreover, we cannot exclude the role of the oxygen radical produced from the dissociation of the adsorbed carbon dioxide over the oxygen vacancies of the yttria-stabilized ZrO_2_ support from participating in the regeneration of the active metallic nickel sites via the reaction with the deposited carbon and adsorbed hydrogen atom produced by the methane decomposition [40].

On the surface of Ni particles, the abstraction of hydrogen from methane takes place, as illustrated in Equations (12)–(17):

(12) $${\mathrm{CH}}_{4}^{*}+\mathrm{Ni}\;\to H/\mathrm{Ni}+{\mathrm{CH}}_{3}^{*}$$

(13) $${\mathrm{CH}}_{3}^{*}+\mathrm{Ni}\;\to 2H/\mathrm{Ni}+{\mathrm{CH}}_{2}^{*}$$

(14) $${\mathrm{CH}}_{2}^{*}+\mathrm{Ni}\;\to 3H/\mathrm{Ni}+{\mathrm{CH}}^{*}$$

(15) $${\mathrm{CH}}^{*}+\mathrm{Ni}\;\to 4H/\mathrm{Ni}+C^{*}$$

(16) $$4H/\mathrm{Ni}\;\to \;2H_{2}+C/\mathrm{Ni}$$

(17) $$C/\mathrm{Ni}+\mathrm{CO}\to {\mathrm{CO}}_{2}+\mathrm{Ni}$$

The deposited C can be gasified and removed from the Ni surface according to Equation (10) or Equation (17) [41,42].

## 4. Conclusions

The study of N_2_-physisorption analysis displayed a type-IV isotherm with H3 hysteresis. The surface areas of Gd_2_O_3_-promoted catalysts were independent of their loading.

In the TPR investigation, the reduction peaks appeared at the moderate-temperature regions, indicating a good interaction between the support and NiO. The addition of Gd_2_O_3_ shifted the diffraction peaks of the support to higher angles, implying the incorporation of the promoter into the unit cell of the YZr support. This fact of Gd_2_O_3_ incorporation was supported by the crystallite size determination of fresh catalysts via Scherrer’s equation, where the un-promoted catalyst presented the lowest value of 16.80 nm, while the crystallite size increased to 17.3 nm for 5Ni+4Gd/YZr and 5Ni+5Gd/YZr and to 17.4 nm for 5Ni+1Gd/YZr, 5Ni+2Gd/YZr, and 5Ni+3Gd/YZr. In the CO_2_-TPD analysis, Gd_2_O_3_-promoted catalysts presented both weak Bronsted basic sites and medium-strength Lewis base sites, unlike the un-promoted one, which exhibited low-intensity weak basic sites.

The Gd_2_O_3_ promotion enhanced catalyst stability by lowering carbon deposition, owing to the dissociative adsorption of methane. The relative carbon resistance of our catalysts could be linked to their basicity endowed by the Gd_2_O_3_ promoter and its ability to sweep off the carbon deposit by gasification reaction via oxycarbonate species. The 5Ni/YZr system showed lower activity than the Gd_2_O_3_-promoted catalysts. The 5Ni+4Gd/YZr catalyst was found to have the highest methane conversion (∼80%), CO_2_ conversion (~86%), and H_2_/CO mole ratio (∼0.90) and the lowest carbon deposit (6.0 wt.%), suggesting that 4.0 wt.% loading was the optimum for the Gd_2_O_3_ promoter. This inspection indicates that Gd_2_O_3_ not only promoted the reaction performance of the catalysts but also contributed to increasing the coke resistance of the catalysts.

## Figures and Tables

**Table 1:** Textural properties (S_BET_, P_v_, and P_d_) of the fresh catalysts.

Catalyst	S_BET_, m^2^/g	P_v_, cm^3^/g	P_d_, nm
5Ni/YZr	31	0.19	24.78
5Ni+1Gd/YZr	27	0.16	23.49
5Ni+2Gd/YZr	26	0.16	24.05
5Ni+3Gd/YZr	27	0.14	21.36
5Ni+4Gd/YZr	26	0.15	22.28
5Ni+5Gd/YZr	27	0.15	21.36

**Table 2:** The shift in the 2θ angle and change in the d-spacing of (111) crystallographic planes of cubic yttria-stabilized zirconia phase for the fresh catalysts.

Catalyst	Gd_2_O_3_ (wt.%)	2θ (°)	d-Spacing for (111), Å	2θ (°)	d-Spacing for (220), Å
5Ni/YZr	0.0	30.04	2.9727	50.15	1.8176
5Ni+1Gd/YZr	1.0	30.04	2.9721	50.16	1.8172
5Ni+2Gd/YZr	2.0	30.05	2.9717	50.17	1.8169
5Ni+3Gd/YZr	3.0	30.07	2.9699	50.18	1.8166
5Ni+4Gd/YZr	4.0	30.11	2.9652	50.19	1.8162
5Ni+5Gd/YZr	5.0	30.19	2.9614	50.24	1.8145

**Table 3:** The shift in the 2θ angle and change in the d-spacing of (111) and (220) crystallographic planes of cubic yttria-stabilized zirconia phase for the spent catalysts.

Catalyst	Gd_2_O_3_ (wt.%)	2θ (°)	d-Spacing for (111), Å	2θ (°)	d-Spacing for (220), Å
5Ni/YZr	0.0	30.00	2.9762	50.02	1.8220
5Ni+1Gd/YZr	1.0	30.15	2.9617	50.24	1.8145
5Ni+2Gd/YZr	2.0	30.32	2.9455	50.49	1.8061
5Ni+3Gd/YZr	3.0	30.09	2.9675	50.14	1.8179
5Ni+4Gd/YZr	4.0	30.66	2.9136	51.00	1.7893
5Ni+5Gd/YZr	5.0	30.27	2.9503	50.40	1.8092

**Table 4:** The comparison of our results with those reported previously in the literature.

Cat	Wt.%		GHSV, L/(h·g)	Rx. Temp., °C	Conversion, %		Mole Ratio		Ref.
	NiO	Gd_2_O_3_			CH_4_	CO_2_	CH_4_/CO_2_	H_2_/CO	
5Ni+1Gd/Al	5	1	29.9	700	83	89	1:1	1	[38]
Gd_0.45_Ni/SiO_2_	6.36	0.52	9.0	750	86.9	75.1	1:0.4	1.42	[37]
3Gd+10Ni/Y_2_O_3_	12.72	3	8	700	84	82	1:1	-	[28]
0.1Gd5NiMCM41	6.36	0.12	39	800	87	91	1:1	0.9	[30]
NiGd_0.45_/SiO_2_	6.36	0.52	9.0	700	67.3	72.4	1:1	-	[39]
5Ni+4Gd/YZr	5	4	42	800	80	86	1:1	0.9	This work

**Table 5:** d-spacing calculated from HRTEM for both fresh and spent 5Ni+4Gd/Yzr.

Catalyst	d-Spacing Calculated from HRTEM, nm	d-Spacing in Bulk YZr, nm	Miller Indices (hkl) Assignment
Fresh	0.248	0.248	411
Spent	0.258	0.263	400

## Data Availability

No data was used for the research described in the article.
